# Acting Out Addictions: Developing Confidence in Addiction Psychiatry Through Simulation-Based Learning

**DOI:** 10.1192/bjo.2026.11310

**Published:** 2026-06-30

**Authors:** Elizabeth Keeper, Maria Rodrigues, Ravinder Hayer, Julia Harrison, Derrett Watts

**Affiliations:** 1 Midlands Partnership NHS Foundation Trust, Stafford, United Kingdom; 2 Birmingham and Solihull Mental Health NHS Foundation Trust, Birmingham, United Kingdom

## Abstract

**Aims:**

Substance misuse and addiction-related problems are routinely encountered within psychiatric services, yet many core psychiatry trainees report limited confidence in assessing and managing alcohol- and opioid-related presentations. Traditional didactic teaching may not adequately prepare trainees for the complexity of real-world clinical encounters. Simulation-based education allows learners to practise challenging scenarios in a controlled setting, while case-based discussions (CBDs) encourage reflective learning using authentic clinical material. This educational initiative aimed to explore whether combining simulation with CBDs could improve trainee confidence in addiction psychiatry.

**Methods:**

Two dedicated addiction psychiatry teaching days were delivered within a regional core psychiatry training programme. Each day consisted of immersive simulation scenarios, structured debriefs, and faculty-facilitated CBDs. Scenarios focused on core clinical skills including substance use history taking, assessment of risk, and management planning for alcohol withdrawal and opioid use. The first session was attended by 12 trainees and the second by 11 trainees (CT1–CT3). In response to feedback, a simulation scenario was revised for the second teaching day to prioritise collaborative discussion of short- and long-term management plans with the patient. Changes in self-reported confidence were measured using pre- and post-session questionnaires, alongside qualitative feedback.

**Results:**

Prior to the first teaching day (n=12), most trainees reported low or neutral confidence in managing addiction-related presentations. Following the session, all trainees reported increased confidence, with the majority describing themselves as somewhat confident and a smaller proportion as extremely confident.

At the second teaching day (n=11), baseline confidence levels were higher, with over one third of trainees reporting some confidence before the session. Post-session responses again demonstrated an upward shift, with most trainees reporting being somewhat or extremely confident. Attendance at both teaching days by some trainees may have contributed to this higher baseline confidence.

Qualitative feedback across both sessions consistently identified simulation as the most impactful learning modality. Trainees valued the opportunity to practise clinical decision-making in real time, make mistakes in a safe environment, and receive immediate, structured feedback. The integration of CBDs was viewed as complementary, supporting consolidation of learning and reflective discussion based on authentic clinical cases.

**Conclusion:**

This combined simulation and CBD teaching programme was associated with improved self-reported confidence. While limited by small participant numbers and reliance on subjective measures, the findings support the continued use of experiential learning approaches. Future iterations will aim to enhance realism through involvement of individuals with lived experience of addiction, with further sessions planned for April 2026.

